# Efficacy of an internet-based tool for improving physician knowledge of chronic kidney disease: an observational study

**DOI:** 10.1186/1471-2369-13-126

**Published:** 2012-09-27

**Authors:** Michelle M Estrella, Stephen D Sisson, Jennifer Roth, Michael J Choi

**Affiliations:** 1 Department of Medicine, Johns Hopkins University School of Medicine, 1830 E. Monument Street, Suite 416, Baltimore, MD 21205, USA; 2 Department of Medicine, Johns Hopkins University School of Medicine, 601 N. Caroline Street, Room 7150 G, Baltimore, MD, 21287, USA

**Keywords:** Kidney disease, Education, Internet, Primary care

## Abstract

**Background:**

Early recognition and management of chronic kidney disease (CKD) are associated with better outcomes. Internal medicine residency should prepare physicians to diagnose and manage CKD.

**Methods:**

To examine whether residency training and program characteristics were associated with CKD knowledge and investigate the effectiveness of an internet-based training module in improving CKD knowledge, we analyzed data from CKD training modules administered annually to U.S. internal medicine residents from July 1, 2005 to June 30, 2009. Baseline CKD knowledge was assessed using pre-tests. The modules’ effectiveness was evaluated by post-tests. Comparisons were performed using *X*^2^ tests and paired t-tests.

**Results:**

Of 4,702 residents, 38%, 33%, and 29% were program year (PGY)-1, PGY-2, and PGY-3, respectively. Baseline CKD knowledge was poor, with mean pre-test scores of 45.1-57.0% across the four years. The lowest pre-test performance was on CKD recognition. Pre-test scores were better with higher training levels (P-trend < 0.001 except 2005–2006 [P-trend = 0.35]). Affiliation with a renal fellowship program or program location within a region of high end-stage kidney disease prevalence was not associated with better baseline CKD knowledge. Completion of the CKD module led to significant improvements from pre- to post-test scores (mean improvement 27.8% [SD: 21.3%] which were consistent from 2005 to 2009.

**Conclusions:**

Knowledge of diagnosis and management of CKD improves during residency training but remains poor among graduating residents. Web-based training can be effective in educating physicians on CKD-related issues. Studies are needed to determine whether knowledge gained from such an intervention translates to improved care of CKD patients.

## Background

An estimated 15 million adults in the U.S have moderate to severe chronic kidney disease (CKD) [1]. A large proportion of these individuals have co-morbid conditions such as diabetes, hypertension, and cardiovascular disease, which often precede the onset of CKD [2]. The culmination of CKD and these accompanying disorders lead to a high rate of hospitalizations, hospitalization-related complications, cardiovascular disease events, and death among these patients. Early recognition and management of CKD can slow the progression towards end-stage kidney disease (ESKD) and may lower risk for other adverse events. To facilitate the recognition and management of CKD by medical care providers, several national and international clinical guidelines and position statements have been published. The care of CKD patients, however, remains highly variable [3]. Among areas in which the provision of renal-focused care prior to dialysis is low, the mortality rate among incident dialysis patients is higher compared to areas with higher proportions of patients who undergo pre-dialysis care.

While early referral to a nephrologist is associated with improved outcomes, the current number of active nephrologists is insufficient to meet the current and anticipated increase in patient workload [4]. Consequently, primary care providers shoulder the burden of managing CKD-related issues in addition to other coexisting chronic medical conditions. Prior studies, however, demonstrate that many primary care providers lack knowledge of existing national CKD guidelines and important aspects of CKD care [5,6]. Residency is a key stage at which CKD training occurs; however, current training leaves many physicians-in-training unfamiliar with CKD recognition and management [7]. One avenue by which this issue may be addressed is web-based medium which affords the use of interactive training tools. In this study, we evaluated the familiarity with recognition and management of CKD among U.S. internal medicine trainees and determined the effectiveness of an existing online-based training module on the diagnosis and management of CKD used by U.S. internal medicine training programs to improve CKD knowledge.

## Methods

### Study design

We conducted a retrospective study of data collected from the CKD training modules administered annually to internal medicine residents from July 1, 2005 to June 30, 2009 as part of their residency curriculum. This online, interactive educational module is available as part of the Johns Hopkins Internet Learning Center ( http://www.hopkinsilc.org). It is utilized by more than 140 U.S. internal medicine training programs and described elsewhere [8]. While some residency programs required the completion of the CKD training module as part of the programs’ ambulatory medicine curriculum, other programs offered the training module electively. This study was approved by the Johns Hopkins Institutional Review Board.

#### Module structure and content development

The CKD didactic module was developed using established principles of curriculum development [9]. Based upon review of published scientific literature and the National Kidney Foundation (NKF) Kidney Disease Outcomes Quality Initiative (KDOQI) guidelines and commentaries, and the Kidney Disease: Improving Global Outcomes (KDIGO) guidelines, key areas of knowledge to inform the focus of the module were identified and encompassed the following: 1) risk factors for CKD; 2) methods for assessment of kidney abnormalities (e.g., albuminuria and proteinuria) and kidney function; 3) recognition and staging of CKD severity; and 4) diagnosis and management of CKD-related conditions including anemia, mineral bone disease, and cardiovascular disease. The module consisted of three sections: 1) a pre-test; 2) a didactic section which can only be accessed upon completion of the pre-test; and 3) a post-test that is provided after completing the didactic section. The pre-test served to assess the user’s CKD knowledge at baseline. The didactic portion of the module consisted of sections to address each of the key knowledge areas. Each didactic section was comprised of questions based on clinical scenarios followed by a written review focused on a key CKD knowledge area. Participants were not re-tested on questions they had answered incorrectly nor were they given additional questions based on incorrect answers. The written review was accompanied by summary tables and flowcharts and references which were electronically linked to PubMed, the NKF KDOQI guidelines and commentaries, and the KDIGO guidelines. Instances in which the scientific literature did not correlate with published guidelines (e.g., target hemoglobin in patients with CKD), information from both sources was conveyed. The post-test re-evaluated the user’s knowledge of CKD upon completion of the didactic section. Participants were able to stop and resume the didactic portion of the module and had no time limitations to complete the pre-test and post-test questions. Participants were informed whether their answer choices were correct and incorrect and were given the correct answer upon completion of the test. These participant feedbacks were accompanied by brief explanations as to why each choice was correct or incorrect. The pre- and post-test questions consisted each of up to 14 multiple choice questions. While the pre- and post-test questions directly inquired about the contents of the CKD didactic module, the paired questions were not the same. The paired pre-test and post-test questions were generated employing established principles of question development [10]. Face and content validity was assessed by having questions reviewed by six faculty clinician-educators familiar with CKD. Questions were revised until all six faculty members agreed that the questions provided adequate information to correctly answer them. Paired pre- and post-test questions were categorized into the following categories: 1) CKD recognition; 2) CKD risk factors; 3) anemia management in CKD; 4) management of CKD-related mineral -bone disorders; and 5) management of hypertension. The didactic module and all questions were reviewed annually by the attending internist and nephrologist for the appropriateness and accuracy of its content. All components were revised annually as needed to reflect significant advances in the CKD scientific literature within the preceding year, to update changes made in the KDOQI and KDIGO guidelines, and to incorporate input from participants’ evaluations of the training tool. The pre-test and post-test questions were again evaluated for face and content validity annually.

#### Study population

Residency programs utilizing the CKD module included primary affiliates of medical schools with substantial funding from the National Institutes of Health and community hospitals [8]. Participants included internal medicine residents and faculty. Participants who completed the pre-test, didactic module, and post-test were considered for inclusion in the study. We excluded individuals who did not complete all three components of the CKD module and who were beyond their third year of post-graduate training.

### Data collection and statistical analysis

Program characteristics were obtained from data collected at the time of a program’s registration. The program characteristics included whether the program was affiliated with a medical school, its geographic location, and the number of residents in each year of training. For programs which participated in the 2008–2009 CKD module, affiliation with an affiliated nephrology fellowship training program was determined through the list of nephrology programs accredited by the Accreditation Council for Graduate Medical Education (ACGME). In addition, programs were delegated to four regions according to varying rates of ESKD available through the U.S. Renal Database System (USRDS) [2]. Data on the average length of time that users took to complete the training module were not collected.

The participants’ familiarity with various CKD topics was evaluated using their pre-test performances. Participants were considered familiar with a particular topic if they had answered the majority of questions on that topic correctly. To assess relative knowledge of chronic disease management, pre-test results from the 2008–2009 CKD module were compared with concurrent pre-test results from a similarly structured didactic module on diabetes. We could not discern if the users of the CKD module were the same as those of the diabetes module. To increase the likelihood of obtaining a comparative user population of the diabetes module, however, we only abstracted pre-test results for the diabetes module from the same programs included in the CKD module for 2008–2009.

Performance data were tabulated for each year and analyzed based on year of training. Additional analyses planned *a priori* included comparisons of pre-test performance data by question category, by affiliation with a nephrology fellowship training program, and by whether the program was located in a region with high versus low ESKD rates. Pre-test scores across categories were compared using the *X*^2^ test, while the pre-test and post-test scores were compared by paired t-tests. Two-sided P-values were calculated. Statistical analyses were performed using Stata/MP, version 11.1 (StataCorp LP, College Station, TX).

## Results

### Program and participant characteristics

Table 1 displays the source population and characteristics of participants included in this study. A total of 5,003 internal medicine residents and attendings completed the CKD module from 2005 to 2009. After excluding individuals who were beyond PGY-3 level of training (n = 301), our study population consisted of 4,702 internal medicine residents, of whom 38%, 33%, and 29% were PGY-1, PGY-2, and PGY-3, respectively. Over the four years examined, the number of programs utilizing the CKD module steadily increased from 23 in 2005 to 87 programs in 2009. The proportions of residents completing the CKD module were relatively similar across levels of training.

**Table 1:** Characteristics of participants of the Internet Learning Center (ILC)

**Characteristics**	**2005-2006**	**2006-2007**	**2007-2008**	**2008-2009**
**No. of programs enrolledin ILC**	23	63	80	87
**No. of ILC registrants**	5596	6920	6136	10,327
**Total no. of participantscompleting CKD module**	560	1322	1435	1686
Population included in currentstudy*				
**No. of programs**	23	61	76	87
**No. of participants (%)**	509	1233	1351	1609
**PGY-1**	161 (32)	466 (36)	503 (37)	698 (43)
** PGY-2**	176 (34)	399 (32)	483 (36)	482 (30)
** PGY-3**	172 (34)	388 (32)	365 (27)	429 (27)
**Programs affiliated with renalfellowship, n (%)**	11 (48)	27 (43)	33 (43)	38 (44)
**Programs by regional ESKDrate, n (%)**				
**Located in region withhighest ESKD rate**	6 (26)	26 (43)	26 (34)	27 (31)
**Located in region withlowest ESKD rate**	1 (4)	6 (10)	4 (5)	9 (10)
**Mean pre-test score,% (SD)**	45.1 (18.2)	49.3 (19.3)	57.0 (20.2)	55.2 (19.8)

*Individuals beyond PGY-3 level of training excluded.

Abbreviations: PGY, post-graduate year; ESKD, end-stage kidney disease; SD, standard deviation.

Of the programs, 9%, 11%, 30% and 50% were located within western, southeastern, midwestern, and northeastern regions of the U.S., respectively. The participating programs ranged in structure from those within community hospital settings to tertiary academic centers. Approximately half of the programs participating in the CKD module were affiliated with a renal fellowship training program, and approximately one-third of the programs were located in regions with the highest prevalence of ESKD in the U.S. Users of this CKD training tool have consistently rated it highly (4.4 out of 5).

### CKD knowledge prior to and after completion of the ckd module

**Figure 1:**
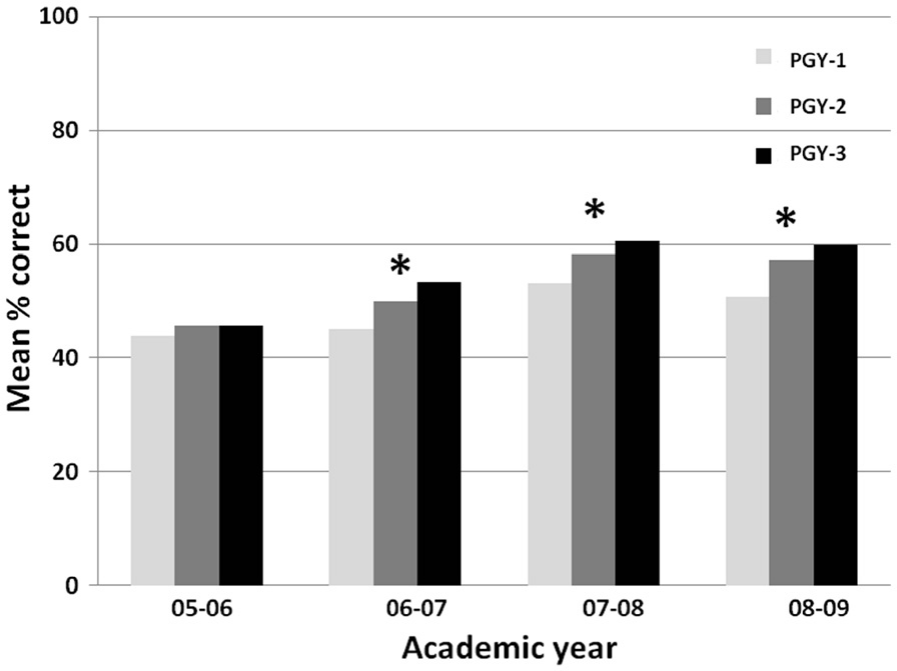
**Baseline CKD knowledge.** This shows the mean pre-test scores among internal medicine residents of various stages of training during the four years that the CKD modules were administered. *P for trend < 0.05.

**Figure 2:**
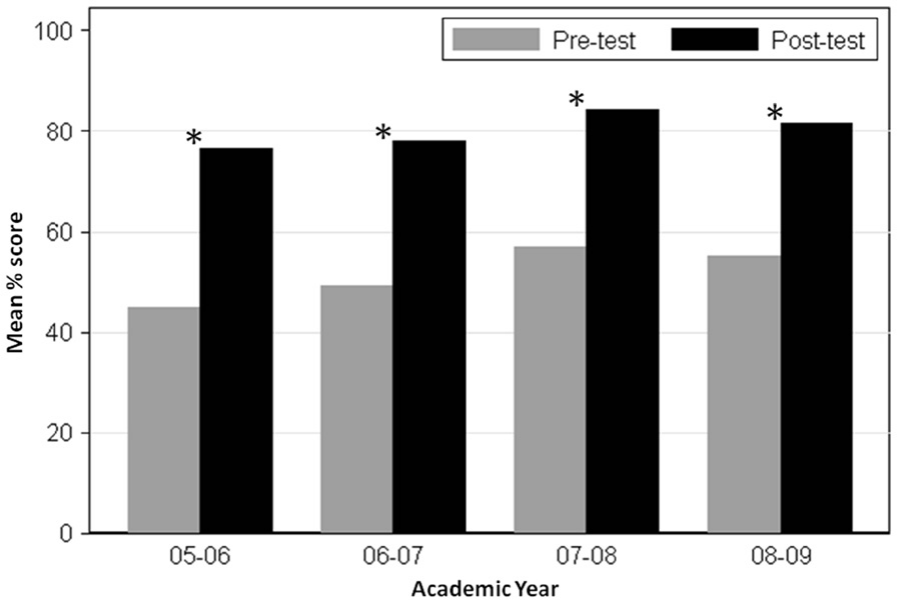
**Improvement in CKD knowledge.** This illustrates the mean pre-test and post-test scores among internal medicine residents during the four years that the CKD modules were administered. *P < 0.05.

Overall, the annual baseline performance on the CKD module was poor, with the mean pre-test scores ranging from 45.1% (standard deviation [SD]: 18.2%) to 57.0% (SD: 20.2%) from 2005 to 2009. We observed a consistent trend towards better scores with higher levels of training; this trend was significant in all years (P for trend <0.001) except 2005–2006 (P for trend =0.35) (Figure 1). With completion of the CKD module, we observed a significant improvement between pre-test and post-test scores (Figure 2). The average increase in scores between the pre-test and post-test was 27.8% (SD: 21.3%). These improvements in performance were consistent across all training levels.

### Subset analyses of data from the 2008–2009 CKD module

A subset analysis of data from the 2008–2009 CKD module showed that baseline knowledge of CKD did not differ by whether the programs were affiliated with a nephrology fellowship program (mean pre-test score: 54.5% for both, P = 0.98). Furthermore, baseline CKD knowledge did not differ between participants whose programs were located in regions with high versus low ESKD rates (mean pre-test score: 50.4% vs. 47.2%, respectively; P = 0.29). Compared with baseline knowledge of the diagnosis and management of diabetes mellitus, baseline CKD knowledge was significantly lower (mean pre-test score: 64.6% vs. 55.2%, respectively; P < 0.001).

The lowest mean pre-test score were for questions pertaining to CKD recognition and staging (53.1%) followed by hypertension management in CKD (56.9%), then mineral bone disease (57.4%). There was, however, a significant increase in scores between the pre-test and post-test within each question subgroup (Table 2). The greatest improvement between pre-test and post-test scores occurred for questions regarding hypertension management in CKD, with a 33.5% increase from the pre-test score.

**Table 2:** **Pre-test and post-test resultsby question topic inthe 2008–2009 data**

**Question topic**	**Pre-test (%)**	**Post-test (%)**	**Change in score betweenpre- and post-test (%)**	**P-value**
**CKD recognition and staging†**	53.1	80.5	+ 27.4	<0.01
**Mineral bone disease**	57.4	80.5	+ 23.1	<0.01
**Hypertension in CKD**	56.9	90.4	+ 33.5	<0.01

Note: Anemia not evaluated due to too few questions.

Abbreviations: CKD, chronic kidney disease.

†CKD recognition and staging includes questions on risk factors for CKD and methods for assessment of kidney abnormalities and function.

## Discussion

Our study demonstrated that only about half of the physicians-in-training were familiar with the diagnosis and management of CKD and its related conditions, significantly lower than residents’ knowledge of diabetes. Baseline knowledge was better with higher levels of post-graduate training. Residents were least familiar with the recognition and staging of CKD severity. Upon completion of the CKD module, however, overall CKD knowledge significantly improved. The knowledge gain was consistent across the key CKD topics examined. To the best of our knowledge, this is the first study to report on a promising, highly feasible, evidence-based online method to enhance residents’ understanding of CKD recognition and management.

Consistent with prior studies, we demonstrated that a large proportion of physicians-in-training were unfamiliar with many aspects of CKD clinical care. In an online survey of U.S. internal medicine residents, Agrawal and colleagues showed that only 54% of respondents recognized persistent proteinuria as CKD and 65% correctly defined CKD stage III [7]. Moreover, nearly one-third of residents were unaware of the KDOQI guidelines. While 82% of their respondents acknowledged mineral bone disease as a potential complication of CKD, only 57% of residents in our study were familiar with the management of CKD mineral bone disease. A survey among U.S. primary care physicians demonstrated that CKD recognition among this group varied depending on the clinical scenario presented. Primary care physicians were less likely to identify CKD among the elderly with serum creatinine values within the reported normal range [5]. In another survey in which a case scenario of a patient with progressive CKD was presented to primary care physicians and nephrologists, Charles *et al.* showed that only 35% of respondents were adherent to clinical testing as recommended by the KDOQI guidelines [6]. In contrast to prior studies, however, we demonstrated the efficacy of an existing training method to improve provider knowledge of CKD and its related issues.

We observed a trend for better performance on the pre-test at higher levels of training, consistent with the study by Agrawal *et al*. [7]. However, these improvements were slight, with an overall 56% pre-test score among PGY-3 despite completing 2 years of residency. These trends may reflect the small gains in CKD knowledge through traditional educational activities and increased clinical experience with progressive training or a cohort of residents repeating the CKD module. Nonetheless, our study underscores ongoing deficits in U.S. trainees with regards to the identification and management of CKD. For example, the residents’ pre-test performance on CKD recognition and management was the poorest among the topics addressed. This is of clinical relevance as early stages of CKD in the general population have been associated with adverse cardiovascular outcomes [11]. Impaired recognition of indicators of CKD, such as albuminuria, may lead to under-recognition of individuals at greater risk of cardiovascular disease. Several factors may account for observed knowledge deficits. Residents may have limited exposure to the care of CKD patients and fewer opportunities to work with nephrologists relative to general internists or other subspecialists. While we were unable to examine these factors specifically in this study, we did not observe differences in performance by the presence or absence of an affiliated nephrology fellowship program or by regional differences in ESKD prevalence. Additional potential factors for knowledge deficits include suboptimal resident attendance at didactic conferences due to competing clinical obligations and a dearth of readily available, concise CKD educational resources. While a systematic review of the impact of the ACGME’s duty-hour restrictions on resident education among other aspects of residency showed conflicting results among studies reviewed [12], a recent survey among 73 residents suggests that strict work hour restrictions may impact the setting in which clinical education occurs, with potentially more residents conducting educational activities at home rather than during work hours [13]. Additional studies, however, are needed to thoroughly evaluate the impact of these factors on resident knowledge of CKD.

Completion of the CKD module was associated with significant improvement in CKD knowledge. Given the increasing scrutiny of resident work-hours and need for flexibility in timing of educational interventions, this CKD module appeared to be feasible for residents to complete. Implementations of online educational tools in other disciplines have shown promising results. A study of 1,786 individuals who used an online educational tool with a similar structure to the CKD module to improve knowledge of cardiac diseases demonstrated a significant improvement between the pre-test and post-test [14]. Similarly, an online educational tool structured like the CKD module was also shown to improve the knowledge of primary care physicians on infectious diseases [15].

Studies assessing the durability of the knowledge gained from internet-based educational activities, however, have shown mixed results. In a study by Casebeer and colleagues, responses to a series of clinical case-based questions from physicians participating in internet-based continuing medical educations activities (CME) were compared to a group of demographically similar physicians who did not partake in these activities [16]. Compared with the latter group, physicians participating in internet-based CME activities were more likely to select evidence-based choices. The questions, however, were administered immediately following the CME activity. The effect of internet-based education on clinical practice was more robustly evaluated in a randomized controlled trial comparing internet-based CME with live interactive CME focused on management of hypercholesterolemia. This study suggested that evidence-based online medical education can lead to durable improvement in knowledge and changes in clinical practice comparable or perhaps better than those from live activities [17]. In this prior study, an audit of the participating clinicians’ charts at 5 months after the intervention showed that a higher proportion of high-risk patients were appropriately treated by physicians randomized to the online intervention versus the live activities (+5% vs. -1.1%, P = 0.04). In contrast, Sangvai and colleagues demonstrated that while an internet web-based module on childhood injury prevention improved performance on post-tests given immediately and 7 months following completion of the module, the module did not impact clinical practice as assessed by videotaped clinical visits. [18] Similar findings were observed in a randomized trial comparing internet-based versus paper-based educational exercises focused on geriatric clinical issues in which neither modality improved clinical practice [19]. These recent studies highlight the difficulty in improving clinical practice and underscore the need for improved educational interventions. Unfortunately in our study, we were unable to track individual participants through their years of residency training due to data collection methods of the Internet Learning Center. As the CKD module was completed at different times across institutions, we were also unable to administer a follow-up test at a more remote period after completion of the module. As such, data were lacking on knowledge gained from the CKD module was retained and improved the clinical care of patients with CKD.

Other potential limitations of our study need consideration. Data on which participants completed the module to fulfill a residency program requirement were not collected nor were data on participants’ time spent on completing the CKD module. Moreover, we did not link individual participant’s rating of the instructive value with that of their performance on the pre- and post-tests. However, we have shown elsewhere using data from all modules offered through the Internet Learning Center that improvements in knowledge are associated with greater learner satisfaction [20]. In aggregate, learners were consistently satisfied with the CKD module, and the high learner satisfaction and improvement in knowledge support the effectiveness of this training modality. Since our study population represented a subset of the Internet Learning Center registrants, our findings may not be applicable to all residency programs and physicians-in-training; however, a range of program types were included in our study. As this study was retrospective, we did not have a comparative control group who received conventional CKD education.

In conclusion, this study presents the first study of an internet-based educational module for improving CKD knowledge. The CKD module appears to be a feasible approach to significantly improving residents’ proficiency in CKD recognition and management. Proponents of internet-based education have highlighted its potential advantages such as ease of access and use, interaction capability, and lower cost [21,22]. Prospective trials, however, are needed to determine whether an internet-based CKD educational module such as ours leads to sustained knowledge of CKD recognition and management and improved care of patients with CKD.

## Conclusions

Knowledge of the diagnosis and management of CKD among residents improves with training but remains suboptimal among residents completing their training. A web-based online training module is effective in educating residents on CKD-related issues whether knowledge acquired from such an intervention leads to improved care of patients with CKD needs further study.

## Abbreviations

ACGME: Accreditation council for graduate medical education; CKD: Chronic kidney disease; ESKD: End-stage kidney disease; KDIGO: Kidney disease: improving global outcomes; KDOQI: Kidney disease outcomes quality initiative; NKF: National kidney foundation; PGY: Post-graduate year; SD: Standard deviation; USRDS: U.S. renal database system.

## Competing interests

Training programs are charged an annual subscription to gain access to the content for their residents. The annual fee was $1500/year, with lesser fees for programs with <30 residents. SDS receives an annual stipend for editorial duties associated with the Internet Learning Center. MME, JR, and MJC have no conflict of interest.

## Authors’ contributions

MME conceived of the study, participated in the design of the study, performed some of the statistical analyses, contributed to the interpretation of the results, and helped draft and revise the manuscript. SDS helped conceive of the study, participated in the design of the study and interpretation of results, and helped revise the manuscript. JR performed some of the statistical analyses and helped revise the manuscript. MJC helped conceive of the study, participated in the design of the study, contributed to the interpretation of results, and helped draft and revise the manuscript. All authors read and approved the final manuscript.

## Authors’ information

MJC serves as the Vice-Chair of Education of the National Kidney Foundation Kidney Disease Outcome Quality Initiative

## Pre-publication history

The pre-publication history for this paper can be accessed here:

http://www.biomedcentral.com/1471-2369/13/126/prepub
